# Supplementary material for: Equity in practice: Assigning competence to shape STEM student participation
Source: PLoS One. 2024 Apr 17;19(4):e0299984. doi: 10.1371/journal.pone.0299984 (PMC11023227; doi:10.1371/journal.pone.0299984)
Supplement: S1 File — (PDF) [file pone.0299984.s004.pdf]

SAN DIEGO STATE UNIVERSITY

Graduate and Research Affairs
Division of Research Affairs San Diego Statue University 5500 Campanile Drive San Diego, CA 92182-1933 Phone 619-594-6622
irb@sdsu.edu

Exempt Amendment Verification

22-Nov-2019

**Principal Investigator:**	**Reinholz, Daniel**
**Co-Principal Investigator:**	**Reinholz, Daniel**
**Department:**	**Math and Statistics**
**Protocol Number:**	**HS-2018-0162**

Title: CAREER: Data Analytics for Equity: Supporting STEM Faculty to Address Implicit Bias in the Classroom (NSF Solicitation: 17-537; Proposal 1943146).

Dear Daniel,

The proposed study amendment was reviewed and verified as exempt in accordance with SDSU's Assurance and federal requirements pertaining to human subjects protections within the Code of Federal Regulations (45 CFR 46.104). This review applies to the following conditions and procedures described in your amendment request:

(1) to change the title to: CAREER: Data Analytics for Equity: Supporting STEM Faculty to Address Implicit Bias in the Classroom (NSF solicitation 17-537; Proposal 1943146)

(2) to increase the number of participants to 100.

The study meets exemption criteria under 45 CFR 46.104(d)(1) and (2)(iii). A limited IRB review was conducted under the provisions at 45 CFR 46.111(a)(7).

The determination of exemption is final and continuing review (Progress Reports) are not required for this study. However, **if any changes to your study are proposed**, you must submit an amendment and receive IRB cerification that the study still meets exemption criteria (per 45 CFR 46.104). Additionally, CITI training must be kept current in order to maintain compliance. Finally, please notify the Human Research Protection Program office at 619-594-6622 or at irb@sdsu.edu if your status as an SDSU-affiliate changes while conducting this research study (you are no longer a SDSU faculty member).

Sincerely,

Rick Gulizia
Director of Research Affairs
San Diego State University
